# National Pension Fund for Nurses

**Published:** 1888-10-06

**Authors:** 


### THE NATIONAL PENSION FUND FOR NURSES.

AT a meeting of the Nottingham and Notts Nursing Association, held on the 1st inst., the following resolution was unanimously passed: "The Nottingham and Notts Nursing Association, as a mark of their appreciation of the benefit likely to accrue to nurses from the establishment of the National Pension Fund for Nurses, are prepared to become annual subscribers of two guineas to the Bonus Fund of that association, and to recommend it to the nurses of their institution." It was stated, in the course of the discussion, that very few nurses have so far remained in the service of this institution for more than four or five years at the outside. A few go elsewhere, but many leave to be married, or give up nursing, or go to nurse sick relatives. Another point of importance was also elicited. It was shown that although many hospitals and institutions look after their nurses during illness, it is only for a limited period. During convalescence this aid generally ceases, and nurses have to depend upon their own resources. In many cases, too, the wages cease during illness. Hence the sickness branch of the Fund was greatly praised, and it was thought that each nurse should enter for 10s. per week sick pay. Only one nurse on the staff at Nottingham is over thirty-five years of age, so all the nurses can join the Fund if they choose on the most favourable terms. We understand the movement in Nottingham in favour of the Pension Fund has been headed by an eminent physician, Dr. Handford, and we hope, with the *British Medical Journal*, that his example may stimulate others all over the country to go and do likewise. We would here remind our readers that Mr. Clifford's interesting paper will be read on Wednesday next at St. Thomas's Hospital, at eight p.m.

THE first number of the *Illustrated Medical News* has just been published. The idea of an illustrated medical paper is excellent, and has been partly carried out by the older professional journals. An illustration is a great aid to teaching, and adds greatly to the interest of description. The present number is well done, and presents a highly interesting and creditable appearance. Its contents are varied, and there is not too much of them, a great desideratum to a tired practitioner at the end of the week. If future numbers prove equal to the first, there is little doubt the new venture will rapidly command success.

